# Pulmonary function in advanced uncomplicated singleton and twin pregnancy* **

**DOI:** 10.1590/S1806-37132014000300007

**Published:** 2014

**Authors:** Anwar Hasan Siddiqui, Nazia Tauheed, Aquil Ahmad, Zehra Mohsin

**Affiliations:** Department of Physiology, Jawaharlal Nehru Medical College, Aligarh, India; Department of Anaesthesiology and Critical Care, Jawaharlal Nehru Medical College, Aligarh, India; Department of Physiology, Jawaharlal Nehru Medical College, Aligarh, India; Department of Obstetrics and Gynaecology, Jawaharlal Nehru Medical College, Aligarh, India

**Keywords:** Respiratory function tests, Respiratory mechanics, Pregnancy, twin, Pregnancy

## Abstract

**Objective:**

Pregnancy brings about significant changes in respiratory function, as evidenced by alterations in lung volumes and capacities, which are attributable to the mechanical impediment caused by the growing foetus. This study was undertaken in order to identify changes in respiratory function during normal pregnancy and to determine whether such changes are more pronounced in twin pregnancy than in singleton pregnancy.

**Methods:**

Respiratory function was assessed in 50 women with twin pregnancies and in 50 women with singleton pregnancies (during the third trimester in both groups), as well as in 50 non-pregnant women. We measured the following pulmonary function test parameters: FVC; FEV_1_; PEF rate; FEV_1_/FVC ratio; FEF_25-75%_; and maximal voluntary ventilation.

**Results:**

All respiratory parameters except the FEV_1_/FVC ratio were found to be lower in the pregnant women than in the non-pregnant women. We found no significant differences between women with twin pregnancies and those with singleton pregnancies, in terms of respiratory function.

**Conclusions:**

Despite its higher physiological demands, twin pregnancy does not appear to impair respiratory function to any greater degree than does singleton pregnancy.

## Introduction

Pregnancy causes many changes in the human body, not all of which are visible. Like other organ systems, the respiratory system, which is highly efficient and sensitive, undergoes profound changes as a result of maternal adaptation to pregnancy. The respiratory system represents the best example of selective adaption of a system during pregnancy.^(1)^ The anatomical and physiological adaptation of the respiratory system in pregnancy must be studied in the interest of properly diagnosing and managing associated respiratory pathologies during pregnancy.^(2)^

In pregnant women, alterations in pulmonary function are attributable to hormonal changes and to the mechanical impediment caused by the growing foetus. In the mucosa of the upper airway, elevated levels of oestrogen cause hyperaemia, hypersecretion, and oedema, leading to nasal obstruction, especially in the third trimester.^(3)^ In addition, progesterone can cause a type of chemoreceptor resetting that results in a slight increase in PaO_2_ and a consequent decrease in PaCO_2_, leading to a state of compensated respiratory alkalosis.^(4)^ The increasing size of the foetus with advancing gestation constitutes a mechanical impediment to the normal process of maternal ventilation. As the uterus expands, there is a ≤ 4 cm cephalad displacement of the diaphragm, with a compensatory increase in the transverse and anteroposterior diameters of the chest, caused by hormonal effects that relax the ligaments.^(5)^

It has been found that tidal volume increases progressively throughout pregnancy, because of increased diaphragm excursion, although inspiratory capacity and vital capacity remain almost unchanged.^(6)^ The increased demand for oxygen without any compensatory increase in the respiratory rate increases the risk of developing maternal hypoxia.

Knowledge of the expected changes in pulmonary parameters is fundamental to the understanding of how any disease state affects pregnancy and vice versa. Pulmonary function tests (PFTs) permit an accurate and reproducible assessment of the functional state of the respiratory system and allow quantification of the severity of lung diseases. This information is also essential for the assessment of whether a patient is a candidate for anaesthesia, as well as of the dangers associated with obstetrical analgesia, given that all of the narcotics and hypnotics used for such analgesia are respiratory depressants.^(7)^

Due primarily to advances in assisted reproductive techniques, the incidence of twin pregnancy has shown a rising trend over the last decade.^(8)^ Because the increased maternal and foetal demands for oxygen are higher in twin pregnancies, we hypothesized that respiratory changes would be more pronounced in twin pregnancy than in singleton pregnancy. In addition, because the uterus is larger in twin pregnancy, the cephalad displacement of the diaphragm might be expected to be greater, as might the laxity of the ligaments of the ribs, both of which could affect lung volumes. Therefore, it seems likely that pregnancy-related changes in respiratory function would be greater in women with twin pregnancy than in those with singleton pregnancy, although that has not been tested. Despite numerous reports of changes in PFT results during pregnancy, not much work has been done on twin pregnancies. The aim of this study was to provide pertinent data by comparing women with twin pregnancies, women with singleton pregnancies, and non-pregnant women, in terms of respiratory function.

## Methods

This was a cross-sectional study involving 40 women with twin pregnancies and 60 women with singleton pregnancies. In all of the pregnant women, respiratory function was assessed at 36 weeks of gestation. In a control group of 50 non-pregnant women, age-matched to the pregnant women, respiratory function was assessed in the first half of the menstrual cycle. The pregnant women were recruited from among those seen at the antenatal clinics of the Department of Obstetrics and Gynaecology at Jawaharlal Nehru Medical College, in the city of Aligarh, India. The controls were volunteers recruited from among the relatives of pregnant women seen at the same antenatal clinics, as well as from among the hospital staff and students. All of the women recruited were between 20 and 32 years of age and had a moderate income, most being homemakers. Of the 40 women with twin pregnancies, 35 were primiparous, as were 48 of the 60 women with singleton pregnancies and 43 of the 50 non-pregnant women. All of the women evaluated were healthy, non-smokers without lung disease, cardiovascular disease, or current respiratory infection. None were taking medication that is believed to alter respiratory function, although some were taking supplemental iron, calcium, or both. Women with acute complications of pregnancy, such as preeclampsia and polyhydramnios, were excluded. The study was approved by the local institutional ethics committee, and all of the participants gave written informed consent. For each subject, a detailed history was taken, a physical examination was performed, and baseline investigations were conducted, in order to rule out cardiorespiratory disease and anaemia.

All PFTs were performed with a computerized spirometer (Medspiror; RMS, Chandigarh, India). Before the PFTs were performed, the procedures were thoroughly explained to the subjects, and the need to maintain an effective seal with the lips around the mouthpiece was emphasized, as was the need to use the nose clip during the procedure. Each subject was instructed to relax for at least 5 min prior to the PFTs.

For each subject, we measured the following parameters: FVC; FEV_1_; FEF_25%-75%_; PEF rate; FEV_1_/FVC ratio; and maximal voluntary ventilation (MVV). All tests were performed in triplicate, and the highest of the three measurements was considered for analysis.

The Kolmogorov-Smirnov test was used in order to assess whether the data were normally distributed. To assess the statistical significance of differences, we employed one-way ANOVA with the Tukey-Kramer post hoc test for multiple comparisons, using the Statistical Package for the Social Sciences, version 17 (SPSS, Inc., Chicago, IL, USA). All normally distributed data are expressed as mean ± standard deviation unless otherwise stated. Medians (with 95% confidence intervals) are used in order to describe skewed data. All analyses were two-tailed, and values of p < 0.05 were considered statistically significant. We calculated that, in order to achieve a power of 80% for the detection of a one standard deviation difference between groups for each measurement, at the 5% level of significance, it would be necessary to recruit at least 16 patients into each group.

## Results

The three groups-twin pregnancy, singleton pregnancy, and control-were comparable on the basis of age, height, weight, blood pressure, and haemoglobin levels (Tables 1 and 2). We observed a significant difference between the study subjects (both groups) and the control subjects in terms of body weight and body mass index. The American Thoracic Society PFT guidelines, established in March of 1991, are based on the height, age, gender, and race of the individual under testing, suggesting that pregnancy-related weight gain has no significant effect on lung function. We found that haemoglobin levels were significantly lower in the twin pregnancy group than in the control group.

**Table 1:** Descriptive statistics of baseline variables in the groups evaluated.a

Variable	Group		
	NP	SP	TP
	(n = 50)	(n = 60)	(n = 40)
Subject age, years	26.72 ± 4.16	26.84 ± 2.95	27.62 ± 3.16
Subject height, cm	154.71 ± 3.11	153.13 ± 2.45	154.45 ± 3.41
Subject weight, kg	54.54 ± 4.92	62.78 ± 5.83	64.78 ± 6.10
Subject BMI, kg/m^2^	22.37 ± 2.80	27.00 ± 3.42	27.13 ± 2.56
Subject SBP, mmHg	118.24 ± 9.14	119.52 ± 9.38	123.63 ± 8.92
Subject DBP, mmHg	77.42 ± 6.52	76.56 ± 5.82	75.24 ± 5.32
Subject haemoglobin, g/dL	11.82 ± 0.54	11.43 ± 0.43	11.29 ± 0.40
Gestational age of foetus, days	-	252 ± 2.52	255 ± 2.17

NP : non-pregnant (control) group

SP : singleton pregnancy group

TP : twin pregnancy group

BMI : body mass index

SBP : systolic blood pressure

DBP : diastolic blood pressure. ^a^Values expressed as mean ± SD

**Table 2:** Results of one-way ANOVA comparing baseline variables between different group pairs.

Variable	NP vs. SP	NP vs. TP	SP vs. TP
	p	p	p
Age	0.346	0.925	0.236
Height	0.244	0.751	0.198
Weight	0.015	0.005	0.061
Body mass index	0.023	0.014	0.138
Systolic blood pressure	0.543	0.064	0.142
Diastolic blood pressure	0.150	0.098	0.248
Haemoglobin	0.175	0.028	0.079

NP : non-pregnant (control) group

SP : singleton pregnancy group

TP : twin pregnancy group

**Figure 1:**
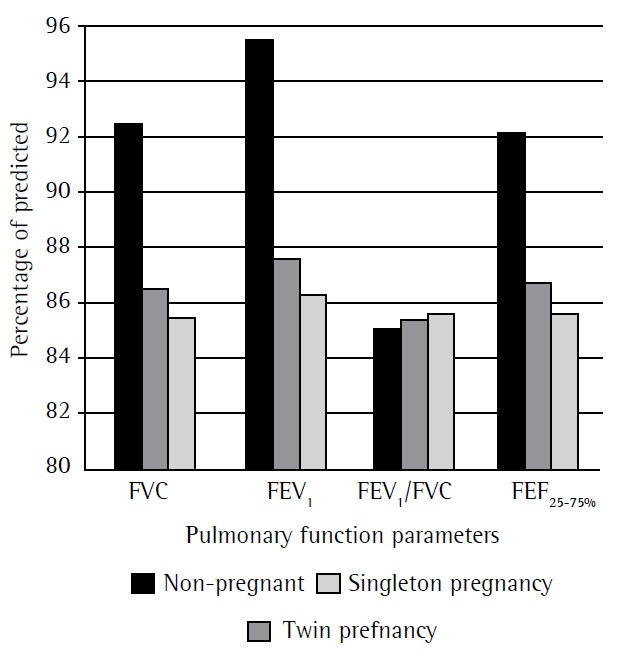
Comparison of pulmonary function parameters among the three groups evaluated.

In the present study, the values for all PFT parameters were lower among the pregnant women (both groups) than among the non-pregnant women (Table 3). Comparisons between various group pairings (Table 4) showed that all of the PFT parameters, with the exception of the FEV_1_/FVC ratio and MVV, were significantly lower for the pregnant women (twin or singleton pregnancy) than for the non-pregnant women. The MVV values were also lower among the pregnant women, although the difference was not significant. As can be seen in Figure 1, there were no significant differences in lung function between the twin and singleton pregnancy groups.

**Table 3:** Descriptive statistics of pulmonary function test results for the groups of women evaluated.a

Variable	Group		
	NP	SP	TP
	(n = 50)	(n = 60)	(n = 40)
FVC			
% of predicted value	92.48 ± 8.43	86.48 ± 4.37	85.56 ± 7.85
Actual value, L	2.64 ± 0.42	2.47 ± 0.29	2.44 ± 0.34
FEV1			
% of predicted value	94.53 ± 6.24	88.64 ± 5.62	86.34 ± 4.39
Actual value, L	2.37 ± 0.18	2.17 ± 0.25	2.14 ± 0.18
FEV1/FVC ratio	85.19 ± 2.61	85.52 ± 2.32	85.73 ± 2.21
FEF25-75%			
% of predicted value	92.12 ± 6.61	86.79 ± 5.76	85.64 ± 6.23
Actual value, L	3.48 ± 0.42	3.21 ± 0.27	3.19 ± 0.34
PEF rate, L/min	417 ± 8.61	313.52 ± 8.05	311.52 ± 6.79
MVV, L/min	104.32 ± 14.45	98.53 ± 13.62	97.68 ± 14.21

NP : non-pregnant (control) group

SP : singleton pregnancy group

TP : twin pregnancy group

MVV : maximal voluntary ventilation. ^a^Values expressed as mean ± SD

**Table 4:** Results of one-way ANOVA comparing pulmonary function test results between different group pairs.

Variable	NP vs. SP	NP vs. TP	SP vs. TP
	p	p	p
FVC	0.013	0.004	0.381
FEV1	0.034	0.029	0.257
FEV1/FVC ratio	0.306	0.321	0.432
PEF rate	0.001	0.001	0.062
FEF25-75%	0.004	0.006	0.247
MVV	0.543	0.477	0.982

NP : non-pregnant (control) group

SP : singleton pregnancy group

TP : twin pregnancy group

MVV : maximal voluntary ventilation

## Discussion

In the present study, we have demonstrated that lung function in the last trimester of pregnancy does not differ significantly between women with twin pregnancies and those with singleton pregnancies. Nevertheless, the values for most respiratory parameters were seen to be significantly lower among pregnant women (twin or singleton pregnancy) than among non-pregnant women.

The decrease in FVC among the pregnant women evaluated in our study can be attributed to the mechanical pressure of the enlarged gravid uterus, which results in the upward displacement of the diaphragm and a consequent restriction of lung mobility. In addition, the elevation of the diaphragm brings about a relative decrease in the negative intrapleural pressure, which hampers forceful expiration.^(9)^ Apart from the mechanical factor, hormonal changes during pregnancy have a significant influence on tracheobronchial smooth muscle tone, the reduction of which can decrease FVC.

Our finding that FEV_1_, FEF_25-75%_, the PEF rate and MVV were lower among the pregnant women might be due to the decline in alveolar PaCO_2_ during pregnancy, which effectively acts as a bronchoconstrictor. Pregnancy is associated with hyperventilation, as the increase in oxygen demand by the growing foetus far exceeds the supply obtained by normal breathing. Hyperventilation in pregnancy is attributed to the effects that progesterone has on the respiratory drive; progesterone not only increases the sensitivity but also reduces the threshold of the respiratory centre.^(10)^ Hyperventilation causes the alveolar PaCO_2_ to drop, resulting in bronchoconstriction. The lower PEF rates and MVV values obtained for the pregnant women evaluated in the present study can also be attributed to the decline in the strength of contraction of the main respiratory muscle (viz., the anterior abdominal muscle) and the internal intercostal muscles during the pregnant state. Studies suggest that this decrease in the muscular force of contraction is due to maternal weight gain, as well as pregnancy-related oedema, altered eating habits, and inadequate nutrition, all of which limit maternal respiratory effort during pregnancy.^(11-13)^ Another factor that might have contributed to lowering the PEF rate and the MVV is the relatively low haemoglobin level observed in the pregnant women. Although none of the subjects had a haemoglobin level < 10 g/dL, even a borderline change in haemoglobin can make a difference.

We found that the FEV_1_/FVC ratio was lower among pregnant women than among non-pregnant women, although the difference was less than significant. That might be because, despite the fact that both FEV_1_ and FVC were lower in the study subjects than in the control subjects, the pregnancy-related decline was not as great for FEV_1_ as it was for FVC.

Our study has at least one limitation. Because the study sample was relatively small, our findings and conclusions might not be generalisable to the general population. A study with a larger sample size might provide more conclusive evidence.

In the present study, the measures of pulmonary function evaluated did not differ significantly between the twin and singleton pregnancy groups. It is known that the decline in alveolar PaCO_2_ during pregnancy increases airway resistance,^(14)^ which is reduced by pregnancy-related increases in the circulating levels of relaxin, progesterone, and cortisol.^(15)^ In twin pregnancy, there might be a balance between these two opposing forces, which would explain why we found no significant differences in comparison with singleton pregnancy. Studies have shown that, in pregnant women, the plasma level of relaxin correlates positively with the number of foetuses.^(15)^ It is evident that the respiratory changes in pregnancy are mediated and determined mainly by the hormonal changes occurring in the body, especially changes in the levels of progesterone and oestrogen. Although twin pregnancy is associated with greater oxygen demand and more uterine distension, the lung volume changes observed in the women with twin pregnancies evaluated in the present study were similar to those seen in the women with singleton pregnancies. This effect can be thought to be mediated by higher levels of progesterone in twin pregnancy. Therefore, we conclude that no significant differences in lung function exist between women with twin pregnancies and those with singleton pregnancies. In healthy women, the respiratory system copes well with the extra demands placed upon it by a twin pregnancy, and no special consideration is required with respect to the dose adjustment of inhalational anaesthetics.
