# Supplementary material for: Pulmonary function in advanced uncomplicated singleton and twin pregnancy
Source: J Bras Pneumol. 2014 May-Jun;40(3):244–9. doi: 10.1590/S1806-37132014000300007 (PMC4109196; doi:10.1590/S1806-37132014000300007)
Supplement: Translation

# Translation: Função pulmonar em mulheres com gestação única ou gemelar avançada e sem complicações*

**Authors:** Anwar Hasan Siddiqui, Nazia Tauheed, Aquil Ahmad, Zehra Mohsin

**Affiliations:** Departamento de Fisiologia, Jawaharlal Nehru Medical College, Aligarh, Índia; Departamento de Anestesiologia e Cuidados Críticos, Jawaharlal Nehru Medical College, Aligarh, Índia; Departamento de Fisiologia, Jawaharlal Nehru Medical College, Aligarh, Índia; Departamento de Obstetrícia e Ginecologia, Jawaharlal Nehru Medical College, Aligarh Índia

**Keywords:** Testes de função respiratória, Mecânica respiratória, Gravidez de gêmeos, Gravidez

## Abstract

**Objetivo:**

A gravidez traz mudanças significativas na função respiratória, evidenciada por alterações nos volumes e capacidades pulmonares, que são atribuíveis ao impedimento mecânico causado pelo feto em crescimento. Este estudo foi realizado a fim de identificar alterações na função respiratória durante a gravidez normal e determinar se tais alterações são mais pronunciadas em gestação gemelar que em gestação única.

**Métodos:**

Foi avaliada a função respiratória de 50 mulheres com gestações gemelares e de 50 mulheres com gestações únicas (durante o terceiro trimestre em ambos os grupos), bem como de 50 mulheres não grávidas. Medimos os seguintes parâmetros de função pulmonar: CVF, FEV1, taxa do PFE, relação VEF_1_/CVF, FEF_25-75%_ e ventilação voluntária máxima.

**Resultados:**

Todos os parâmetros, exceto a relação VEF_1_/CVF, foram menores nas mulheres grávidas do que nas mulheres não grávidas.Não foram encontradas diferenças significativas entre as mulheres com gestações gemelares e aquelas com gestações únicas em relação à função respiratória.

**Conclusões:**

Apesar das demandas fisiológicas maiores da gestação gemelar, essa não parece causar um comprometimento maior da função respiratória do que a gestação única.

## Introdução

A gravidez causa muitas mudanças no corpo humano, nem todas visíveis. Como outros sistemas orgânicos, o sistema respiratório, que é altamente eficiente e sensível, passa por mudanças profundas como resultado da adaptação materna à gravidez. O sistema respiratório representa o melhor exemplo de adaptação seletiva de um sistema durante a gravidez.^(^ ^1^ ^)^ A adaptação anatômica e fisiológica do sistema respiratório na gravidez deve ser estudada com vistas ao diagnóstico e manejo corretos de doenças respiratórias associadas durante a gravidez.^(^ ^2^ ^)^

Em mulheres grávidas, as alterações na função pulmonar são atribuíveis às alterações hormonais e ao impedimento mecânico causado pelo feto em crescimento. Na mucosa das vias aéreas superiores, níveis elevados de estrogênio causam hiperemia, hipersecreção e edema, levando a obstrução nasal, especialmente no terceiro trimestre.^(^ ^3^ ^)^ Além disso, a progesterona pode causar um tipo de reajuste dos quimiorreceptores que resulta em leve aumento da PaO_2_ e consequente diminuição da PaCO_2_, levando a um estado de alcalose respiratória compensada.^(^ ^4^ ^)^ O tamanho crescente do feto com o avanço da gestação constitui um impedimento ao processo normal da ventilação materna. Conforme o útero se expande, há um deslocamento cefálico do diafragma ( ≤ 4 cm), com aumento compensatório dos diâmetros transversal e anteroposterior do tórax, causado por efeitos hormonais que relaxam os ligamentos.^(^ ^5^ ^)^

Constatou-se que o volume corrente aumenta progressivamente durante toda a gravidez, em função do aumento da excursão diafragmática, embora a capacidade inspiratória e a capacidade vital permaneçam quase inalteradas.^(^ ^6^ ^)^ O aumento da demanda de oxigênio sem nenhum aumento compensatório da frequência respiratória aumenta o risco de hipóxia materna.

O conhecimento das alterações esperadas nos parâmetros pulmonares é fundamental para a compreensão de como qualquer estado de doença afeta a gravidez e vice-versa. Os testes de função pulmonar (TFP) permitem uma avaliação precisa e reprodutível do estado funcional do sistema respiratório e possibilitam a quantificação da gravidade das doenças pulmonares. Essas informações também são essenciais para avaliar se um paciente é candidato a anestesia, bem como para avaliar os perigos associados à analgesia obstétrica, visto que todos os narcóticos e hipnóticos utilizados para essa analgesia são depressores respiratórios.^(^ ^7^ ^)^

Devido principalmente aos avanços nas técnicas de reprodução assistida, a incidência de gestação gemelar tem mostrado uma tendência crescente ao longo da última década.^(^ ^8^ ^)^ Como o aumento das demandas maternas e fetais por oxigênio é maior nas gestações gemelares, levantamos a hipótese de que as alterações respiratórias seriam mais pronunciadas na gestação gemelar do que na gestação única. Além disso, como o útero é maior na gestação gemelar, pode-se supor que o deslocamento cefálico do diafragma seja maior, bem como o relaxamento dos ligamentos das costelas, ambos os quais podem afetar os volumes pulmonares. Portanto, parece provável que as alterações na função respiratória relacionadas à gestação sejam maiores em mulheres com gestação gemelar do que naquelas com gestação única, embora isso não tenha sido testado. Apesar de inúmeros relatos de alterações em resultados de TFP durante a gravidez, não há muitas pesquisas sobre gestações gemelares. O objetivo deste estudo foi fornecer informações pertinentes obtidas pela comparação da função respiratória entre mulheres com gestações gemelares, mulheres com gestações únicas e mulheres não grávidas.

## Métodos

Trata-se de um estudo de corte transversal com 40 mulheres com gestações gemelares e 60 mulheres com gestações únicas. Em todas as mulheres grávidas, a função respiratória foi avaliada na 36ª semana de gestação. Em um grupo controle de 50 mulheres não grávidas, pareadas por idade com as mulheres grávidas, a função respiratória foi avaliada na primeira metade do ciclo menstrual. As mulheres grávidas foram recrutadas das clínicas de atendimento pré-natal do Departamento de Obstetrícia e Ginecologia da Jawaharlal Nehru Medical College, na cidade de Aligarh, Índia. Os controles eram voluntárias recrutadas entre as parentes das mulheres grávidas assistidas nas mesmas clínicas de atendimento pré-natal, bem como entre as funcionárias e estudantes do hospital. Todas as mulheres recrutadas tinham entre 20 e 32 anos de idade e possuíam renda moderada, sendo a maioria do lar. Das 40 mulheres com gestações gemelares, 35 eram primíparas, assim como o eram 48 das 60 mulheres com gestações únicas e 43 das 50 mulheres não grávidas. Todas as mulheres avaliadas eram não fumantes saudáveis que não apresentavam doenças pulmonares ou cardiovasculares, nem infecções respiratórias atuais. Nenhuma estava em uso de medicação que se acredita que altere a função respiratória, embora algumas estivessem em uso de suplementação de ferro e/ou cálcio. Foram excluídas as mulheres com complicações agudas da gestação, tais como pré-eclampsia e polidrâmnio. O estudo foi aprovado pelo comitê de ética institucional local, e todas as participantes assinaram um termo de consentimento livre e esclarecido. Para cada sujeito, realizaram-se anamnese detalhada, exame físico e investigações iniciais, a fim de descartar doenças cardiorrespiratórias e anemia.

Todos os TFP foram realizados com um espirômetro computadorizado (Medspiror; RMS, Chandigarh, Índia). Antes de os TFP serem realizados, os procedimentos foram amplamente explicados aos sujeitos, e enfatizou-se a necessidade de manter uma vedação eficaz com os lábios ao redor do bocal, bem como a necessidade do uso do clipe nasal durante o procedimento. Cada sujeito foi instruído a relaxar por pelo menos 5 min antes dos TFP.

Para cada sujeito, medimos os seguintes parâmetros: CVF; VEF_1_; FEF25%-75%; taxa do PFE; relação VEF_1_/CVF; e ventilação voluntária máxima (VVM). Todos os testes foram realizados em triplicata, sendo considerada para a análise a maior das três medidas.

O teste de Kolmogorov-Smirnov foi utilizado para determinar se os dados tinham distribuição normal. Para determinar a significância estatística das diferenças, empregamos a ANOVA de um fator com o teste *post hoc* de Tukey-Kramer para comparações múltiplas, utilizando o *Statistical Package for the Social Sciences*, versão 17 (SPSS, Inc., Chicago, IL, EUA). Todos os dados que apresentaram distribuição normal foram expressos em média ± desvio padrão, a menos que indicado de outra forma. Medianas (com intervalos de confiança de 95%) foram utilizadas para descrever dados com viés. Todas as análises foram bicaudais, e valores de p < 0,05 foram considerados estatisticamente significativos. Calculamos que, para a obtenção de um poder de 80% para detectar uma diferença de um desvio padrão entre os grupos para cada medição, em um nível de significância de 5%, seria necessário recrutar pelo menos 16 pacientes para compor cada grupo.

## Resultados

Os três grupos - mulheres com gestação gemelar, mulheres com gestação única e controle - eram comparáveis em termos de idade, estatura, peso, pressão arterial e níveis de hemoglobina (Tabelas 1 e 2). Observou-se uma diferença significativa entre os sujeitos dos grupos estudo (ambos os grupos) e os sujeitos do grupo controle em relação ao peso corporal e ao índice de massa corpórea. As diretrizes para TFP estabelecidas pela *American Thoracic Society* em março de 1991 se baseiam na estatura, idade, sexo e raça do indivíduo em teste, sugerindo que o ganho de peso relacionado à gestação não tem efeito significativo sobre a função pulmonar. Constatou-se que os níveis de hemoglobina foram significativamente menores no grupo de mulheres com gestação gemelar do que no grupo controle.

**Tabela 1:** Análise estatística descritiva das variáveis iniciais nos grupos avaliados.

Variável	Grupo		
	NG	GU	GG
	(n = 50)	(n = 60)	(n = 40)
Idade do sujeito (anos), média ± dp	26,72 ± 4,16	26,84 ± 2,95	27,62 ± 3,16
Estatura do sujeito (cm), média ± dp	154,71 ± 3,11	153,13 ± 2,45	154,45 ± 3,41
Peso do sujeito (kg), média ± dp	54,54 ± 4,92	62,78 ± 5,83	64,78 ± 6,10
IMC do sujeito, média ± dp	22,37 ± 2,80	27,00 ± 3,42	27,13 ± 2,56
PAS do sujeito (mmHg), média ± dp	118,24 ± 9,14	119,52 ± 9,38	123,63 ± 8,92
PAD do sujeito (mmHg) , média ± dp	77,42 ± 6,52	76,56 ± 5,82	75,24 ± 5,32
Hemoglobina do sujeito (g/dL), média ± dp	11,82 ± 0,54	11,43 ± 0,43	11,29 ± 0,40
Idade gestacional do feto (dias), média ± dp	-	252 ± 2,52	255 ± 2,17

NG : grupo de mulheres não grávidas (controle)

GU : grupo de mulheres com gestação única

GG : grupo de mulheres com gestação gemelar

IMC : índice de massa corpórea

PAS : pressão arterial sistólica

PAD : pressão arterial diastólica.

**Tabela 2:** Resultados da ANOVA de um fator comparando as variáveis iniciais entre diferentes pares de grupos.

Variável	NG vs. GU	NG vs. GG	GU vs. GG
	p	p	p
Idade	0,346	0,925	0,236
Estatura	0,244	0,751	0,198
Peso	0,015	0,005	0,061
Índice de massa corpórea	0,023	0,014	0,138
Pressão arterial sistólica	0,543	0,064	0,142
Pressão arterial diastólica	0,150	0,098	0,248
Hemoglobina	0,175	0,028	0,079

NG : grupo de mulheres não grávidas (controle)

GU : grupo de mulheres com gestação única

GG : grupo de mulheres com gestação gemelar

No presente estudo, os valores para todos os parâmetros dos TFP foram menores entre as mulheres grávidas (ambos os grupos) do que entre as mulheres não grávidas (Tabela 3). As comparações entre vários pares de grupos (Tabela 4) mostraram que todos os parâmetros dos TFP, exceto a relação VEF_1_/CVF e a VVM, foram significativamente menores para as mulheres grávidas (gestação gemelar ou única) do que para as mulheres não grávidas. Os valores da VVM também foram menores entre as mulheres grávidas, embora a diferença não tenha sido significativa. Como se pode observar na Figura 1, não houve diferenças significativas na função pulmonar entre o grupo de mulheres com gestação gemelar e o grupo de mulheres com gestação única.

**Tabela 3:** Análise estatística descritiva dos resultados dos testes de função pulmonar para os grupos avaliados.

Variável	Grupo		
	NG	GU	GG
	(n = 50)	(n = 60)	(n = 40)
CVF			
% do valor previsto, média ± dp	92,48 ± 8,43	86,48 ± 4,37	85,56 ± 7,85
Valor real (L), média ± dp	2,64 ± 0,42	2,47 ± 0,29	2,44 ± 0,34
VEF1			
% do valor previsto, média ± dp	94,53 ± 6,24	88,64 ± 5,62	86,34 ± 4,39
Valor real (L), média ± dp	2,37 ± 0,18	2,17 ± 0,25	2,14 ± 0,18
Relação VEF1/CVF	85,19 ± 2,61	85,52 ± 2,32	85,73 ± 2,21
FEF25-75%			
% do valor previsto, média ± dp	92,12 ± 6,61	86,79 ± 5,76	85,64 ± 6,23
Valor real (L), média ± dp	3,48 ± 0,42	3,21 ± 0,27	3,19 ± 0,34
Taxa do PFE (L/min), média ± dp	417 ± 8,61	313,52 ± 8,05	311,52 ± 6,79
VVM (L/min), média ± dp	104,32 ± 14,45	98,53 ± 13,62	97,68 ± 14,21

NG : grupo de mulheres não grávidas (controle)

GU : grupo de mulheres com gestação única

GG : grupo de mulheres com gestação gemelar

VVM : ventilação voluntária máxima

**Tabela 4:** Resultados da ANOVA de um fator comparando os resultados dos testes de função pulmonar entre diferentes pares de grupos.

Variável	NG vs. GU	NG vs. GG	GU vs. GG
	p	p	p
CVF	0,013	0,004	0,381
VEF1	0,034	0,029	0,257
Relação VEF1/CVF	0,306	0,321	0,432
Taxa do PFE	0,001	0,001	0,062
FEF25-75%	0,004	0,006	0,247
VVM	0,543	0,477	0,982

NG : grupo de mulheres não grávidas (controle)

GU : grupo de mulheres com gestação única

GG : grupo de mulheres com gestação gemelar

VVM : ventilação voluntária máxima

**Figura 1:** Comparação dos parâmetros de função pulmonar entre os três grupos avaliados.

## Discussão

No presente estudo, demonstramos que a função pulmonar no último trimestre da gestação não difere significativamente entre as mulheres com gestações gemelares e aquelas com gestações únicas. No entanto, os valores para a maioria dos parâmetros respiratórios se mostraram significativamente menores entre as mulheres grávidas (gestação gemelar ou única) do que entre as mulheres não grávidas.

A diminuição da CVF entre as mulheres grávidas avaliadas em nosso estudo pode ser atribuída à pressão mecânica do útero gravídico aumentado, que resulta no deslocamento do diafragma para cima e consequente restrição da mobilidade pulmonar. Além disso, a elevação do diafragma acarreta uma diminuição relativa da pressão negativa intrapleural, o que dificulta a expiração forçada.^(^ ^9^ ^)^ Além do fator mecânico, as alterações hormonais durante a gravidez têm uma influência significativa sobre o tônus da musculatura lisa brônquica, e a redução desse tônus pode diminuir a CVF.

Nosso achado de que o VEF_1_, o FEF_25-75%_, a taxa do PFE e a VVM foram menores entre as mulheres grávidas pode se dever ao declínio da PaCO_2_ alveolar durante a gravidez, que efetivamente age como um broncoconstritor. A gravidez está associada à hiperventilação, uma vez que o aumento da demanda de oxigênio pelo feto em crescimento excede em muito a oferta obtida com a respiração normal. A hiperventilação na gravidez é atribuída aos efeitos da progesterona sobre o drive respiratório; a progesterona não só aumenta a sensibilidade, mas também reduz o limiar do centro respiratório.^(^ ^10^ ^)^ A hiperventilação faz com que a PaCO_2_ caia, resultando em broncoconstrição. As taxas do PFE e valores da VVM mais baixos obtidos para as mulheres grávidas avaliadas no presente estudo também podem ser atribuídos ao declínio da força de contração da musculatura respiratória principal (a saber, a musculatura abdominal anterior) e da musculatura intercostal interna durante o estado de gravidez. Estudos sugerem que essa diminuição da força de contração muscular se deve ao ganho de peso materno, bem como a edema relacionado à gestação, hábitos alimentares alterados e nutrição inadequada, todos os quais limitam o esforço respiratório materno durante a gravidez.^(^ ^11^ ^-^ ^13^ ^)^ Outro fator que pode ter contribuído para a redução da taxa do PFE e a redução da VVM é o nível relativamente baixo de hemoglobina observado nas mulheres grávidas. Embora nenhum dos sujeitos tenha apresentado um nível de hemoglobina < 10 g/dL, até mesmo uma alteração marginal na hemoglobina pode fazer diferença.

Constatamos que a relação VEF_1_/CVF foi menor entre as mulheres grávidas do que entre as mulheres não grávidas, embora a diferença não tenha sido significativa. Isso pode ter ocorrido porque, apesar do fato de que tanto o VEF_1_ quanto a CVF foram menores nos sujeitos dos grupos estudo do que nos sujeitos do grupo controle, o declínio relacionado à gestação não foi tão grande para o VEF_1_ quanto para a CVF.

Nosso estudo tem pelo menos uma limitação. Como a amostra do estudo foi relativamente pequena, nossos achados e conclusões podem não ser generalizáveis para a população em geral. Um estudo com uma amostra maior pode fornecer evidências mais conclusivas.

No presente estudo, as medidas de função pulmonar avaliadas não diferiram significativamente entre o grupo de mulheres com gestação gemelar e o grupo de mulheres com gestação única. Sabe-se que o declínio da PaCO_2_ alveolar durante a gravidez aumenta a resistência das vias aéreas,^(^ ^14^ ^)^ que é reduzida por aumentos dos níveis circulantes de relaxina, progesterona e cortisol relacionados à gestação.^(^ ^15^ ^)^ Na gestação gemelar, pode haver um equilíbrio entre essas duas forças opostas, o que explicaria porque não encontramos diferenças significativas na comparação com a gestação única. Estudos têm demonstrado que, nas mulheres grávidas, os níveis plasmáticos de relaxina apresentam correlação positiva com o número de fetos.^(^ ^15^ ^)^ É evidente que as alterações respiratórias na gravidez são mediadas e determinadas principalmente pelas alterações hormonais que ocorrem no corpo, especialmente as alterações nos níveis de progesterona e estrogênio. Embora a gestação gemelar esteja associada a maior demanda de oxigênio e maior distensão uterina, as alterações no volume pulmonar observadas nas mulheres com gestações gemelares avaliadas no presente estudo foram semelhantes às vistas nas mulheres com gestações únicas. Acredita-se que esse efeito possa ser mediado por níveis maiores de progesterona na gestação gemelar. Portanto concluímos que não existem diferenças significativas na função pulmonar entre mulheres com gestações gemelares e aquelas com gestações únicas. Em mulheres saudáveis, o sistema respiratório lida bem com as demandas extras que lhe são impostas por uma gestação gemelar, e nenhuma consideração especial é necessária no que diz respeito ao ajuste da dose de anestésicos inalatórios.
